# A Case of Rendezvous Dilation under Laparotomy for Pancreaticojejunostomy Stricture after Pancreaticoduodenectomy

**DOI:** 10.70352/scrj.cr.25-0042

**Published:** 2025-05-12

**Authors:** Masaharu Ishida, Shimpei Maeda, Shuichiro Hayashi, Shingo Yoshimachi, Hideaki Sato, Akiko Kusaka, Mitsuhiro Shimura, Shuichi Aoki, Masahiro Iseki, Daisuke Douchi, Takayuki Miura, Masamichi Mizuma, Kiyoshi Kume, Atsushi Masamune, Takashi Kamei, Michiaki Unno

**Affiliations:** 1 Department of Surgery, Tohoku University Graduate School of Medicine, Sendai, Miyagi, Japan; 2 Division of Gastroenterology, Tohoku University Graduate School of Medicine, Sendai, Miyagi, Japan

**Keywords:** case report, pancreaticojejunostomy, pancreaticoduodenectomy, anastomotic stenosis, rendezvous

## Abstract

**INTRODUCTION:**

Pancreatico-enterostomy stenosis is a late postoperative complication following pancreaticoduodenectomy. We report a case in which a surgical “rendezvous” procedure was performed to address the stenosis.

**CASE PRESENTATION:**

A 20-year-old woman underwent laparoscopic pancreaticoduodenectomy for a solid pseudopapillary neoplasm in the pancreatic head. During the follow-up, she presented with recurrent abdominal pain, elevated pancreatic enzymes, and dilation of the main pancreatic duct, suggestive of a remnant pancreatitis secondary to pancreaticojejunostomy stenosis. Endoscopic evaluation using double-balloon endoscopy failed to locate the anastomosis. Endoscopic ultrasound enabled puncture and cannulation of the main pancreatic duct, though the anastomotic site remained obstructed. An endoscopic nasal pancreatic drainage tube was placed within the main pancreatic duct. Subsequently, open surgery was performed to dilate the anastomosis and insert a drainage tube. A gastrotomy was created, revealing a fistula between the stomach and pancreas, and a guidewire was introduced from the fistula to the anastomosis. The guidewire was inserted through the fistula and guided to the jejunum through the anastomosis by incising the jejunum on the opposite side of the pancreaticojejunostomy. A stent was deployed across the anastomosis, and a transgastric pancreatic duct drainage was made. The patient subsequently underwent endoscopic dilation and is currently asymptomatic.

**CONCLUSIONS:**

Symptomatic anastomotic stenosis necessitates treatment, with an endoscopic approach generally preferred as the first-line option. When endoscopic visualization of the anastomosis proves challenging, an ultrasound endoscope can be utilized to puncture the main pancreatic duct from the stomach and establish a connection to the jejunum (the “rendezvous” method). If endoscopic interventions are unsuccessful, surgical intervention is warranted. Surgical management often involves anastomotic resection and reanastomosis. In this case, a less invasive surgical “rendezvous” approach was successfully employed, which may offer a valuable surgical alternative for managing pancreatico-enterostomy stenosis after pancreaticoduodenectomy.

## Abbreviations

ENPD
: endoscopic nasal pancreatic drainage
EUS
: ultrasound endoscope
EUS-PD
: endoscopic ultrasound-guided pancreatic duct drainage
LPD
: laparoscopic pancreaticoduodenectomy
MRCP
: magnetic resonance cholangiopancreatography

## INTRODUCTION

In pancreaticoduodenectomy, or Whipple procedure, anastomosis is performed between the pancreas and digestive tract to drain the pancreatic juice. Anastomotic stenosis is a late complication of pancreatico-enterostomy, which leads to pancreatitis due to impaired pancreatic juice outflow and reduced nutritional status due to exocrine pancreatic insufficiency. For an anastomotic stenosis of the pancreaticojejunostomy, reoperation was previously recommended,^1)^ but now endoscopic intervention is performed, with the anastomosis confirmed endoscopically using balloon dilation or stent placement at the stenosis. Lithotomy is also performed if pancreatic stones are present. In cases where it is difficult to identify the anastomosis endoscopically, the main pancreatic duct of the remnant pancreas is punctured from the stomach under endoscopic ultrasound, and a catheter is guided through the anastomosis to merge with the endoscope in the jejunum (rendezvous),^2)^ or a method is adapted in which only pancreatic duct drainage is performed when the “rendezvous” is difficult EUS-PD. Surgical treatment is selected when conservative or endoscopic treatment is ineffective,^3)^ and resection and reconstruction of the pancreaticojejunostomy are often performed.

In this study, we encountered a case in which EUS-PD was performed, followed by a surgical “rendezvous” procedure. Our method may be an effective approach for treating stenosis after pancreaticoduodenectomy.

## CASE PRESENTATION

In January 2018, a 20-year-old female patient underwent LPD for a solid pseudopapillary neoplasm in the head of the pancreas. LPD was performed at our hospital, with pancreaticojejunostomy reconstructed using the modified Blumgart technique.^4)^ At the time of LPD, the pancreas was soft, and the diameter of the pancreatic duct was 8 mm. An external stent of the pancreatic duct was employed during the surgery and removed 2 weeks later. The patient developed a surgical site infection and mild pancreatitis but improved conservatively and was discharged on the 52nd postoperative day. Postoperatively, the patient’s amylase level normalized, although a mild elevation in lipase was observed.

**Fig. 1:**
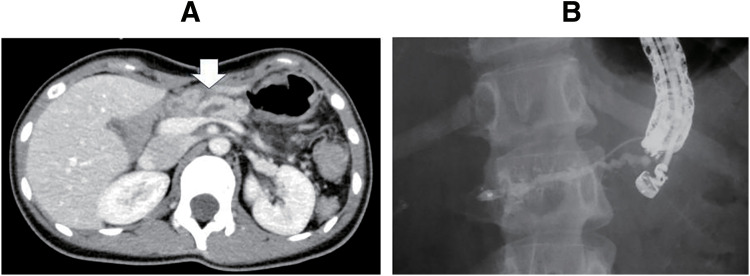
Preoperative imaging findings. The patient experienced repeated abdominal pain due to pancreatitis after LPD for solid pseudopapillary neoplasm of the pancreatic head. A CT scan revealed a dilated main pancreatic duct, indicated by the arrow (**A**). Since the anastomosis could not be identified by endoscopy, the main pancreatic duct was punctured under endoscopic ultrasound (**B**). The guidewire could not pass through the anastomosis, and an endoscopic nasal pancreatic drainage tube was placed in the main pancreatic duct. LPD, laparoscopic pancreaticoduodenectomy

Four months after the LPD, she presented with recurrent abdominal pain and elevated pancreatic enzymes, specifically both amylase and lipase, as well as dilation of the main pancreatic duct (**Fig. 1A**), suggesting remnant pancreatitis secondary to pancreaticojejunostomy stenosis. An endoscopic approach to the pancreaticojejunostomy was performed using double-balloon endoscopy, but the anastomosis could not be identified. Since the dilated main pancreatic duct was identified using an EUS, the main pancreatic duct was punctured from the stomach, and a cannula was inserted using EUS. The main pancreatic duct was interrupted at the anastomotic site, and the jejunum could not be imaged. An attempt was made to pass the anastomotic stenosis, but it was unsuccessful. An ENPD tube was put in place, and the procedure was completed using EUS-PD (**Figs. 1B** and **2A**) in June 2018 (5 months after LPD).

**Fig. 2:**
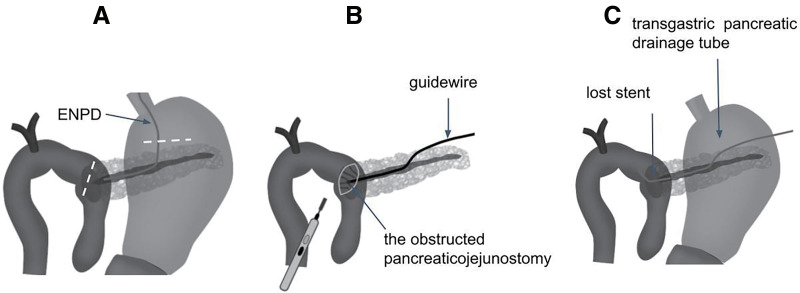
Surgical procedures. Gastrotomy and jejunotomy (indicated by white dashed lines) were performed (**A**). A guidewire was introduced through the fistula between the stomach and the pancreas, but it could not pass through the obstructed pancreaticojejunostomy. The obstructed anastomosis was incised by electric cautery, and then the guidewire was led into the elevated jejunum through the anastomosis (**B**). A lost stent was placed through the anastomosis, and the transgastric pancreatic drainage was also put in place (**C**). ENPD, endoscopic nasal pancreatic drainage

**Fig. 3:**
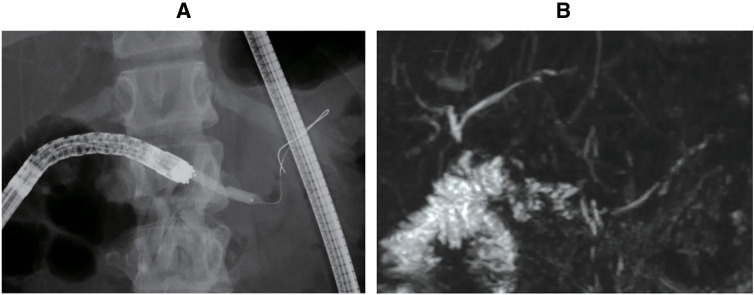
Postoperative imaging findings. After the operation, endoscopic dilation was performed at the anastomosis site (**A**). Currently, there are no signs of pancreatic duct dilation (**B**) and no symptoms of abdominal pain.

Two weeks later, an open surgery was performed to dilate the anastomosis and place a drainage tube through it. During the surgery, under gastrotomy, the fistula between the stomach and pancreas was identified along the ENPD tube. A guidewire was introduced from the fistula to the anastomosis, but it could not pass through the obstructed pancreaticojejunostomy. The opposite side of the anastomosis of the elevated jejunum was incised, and the guidewire from the fistula was confirmed by ultrasound and palpation. The stenosed anastomosis was incised using electric cautery (**Fig. 2B**), and then the guidewire was led into the elevated jejunum through the anastomosis. A lost stent was placed at the anastomosis via the guidewire after dilating the anastomosis. A pancreatic duct drainage was placed transgastrically in the ENPD that had originally been placed, and then guided outside the body through the abdominal wall (**Fig. 2C**). The patient was discharged without any complications after the surgery. The external drainage and the lost stent were removed in 2 and 6 months, respectively. The pancreaticojejunostomy site was subsequently identified by endoscopy, and endoscopic dilation was successfully performed (**Fig. 3A**). For 6 years since the surgery, the dilation of the main pancreatic duct has improved (**Fig. 3B**), and there is currently no evidence of dilation, nor have there been any episodes of pancreatitis.

## DISCUSSION

When discussing anastomotic stenosis after pancreaticoduodenectomy, the definition and scope of the condition should first be clarified; however, in reality, it is not easy to determine what constitutes anastomotic stenosis.

The concept of anastomotic stenosis is that, morphologically, there is a certain degree of difference in the diameter of the main pancreatic duct of the remnant pancreas and the anastomosis, and functionally, there is an obstruction to the outflow of pancreatic juice. Possible functional evaluations include measuring the amount of pancreatic juice and pancreatic duct pressure. One method of evaluation is to take MRCP images after the administration of secretin.^5)^ Because evaluation is difficult using only CT, which is routinely performed as a postoperative examination, MRCP^6)^ and endoscopic findings are considered important for confirming anastomotic stenosis. However, if there are no clinical symptoms such as abdominal pain, and no findings of pancreatitis or coexisting pancreatic stones found on CT scans, it is unlikely that further MRCP or endoscopic examinations will be performed. As a result, anastomotic stenosis often remains undetected.

The follow-up period also has a significant impact on the determination of anastomotic stenosis. Some reports have shown that the rate of anastomotic stenosis 1 year after surgery is 6%,^7)^ but other reports have shown that the median time until anastomotic stenosis occurs is more than 3 years.^8)^ Therefore, long-term follow-up is necessary to determine anastomotic stenosis.

Kurosaki et al. reported that approximately 30% of cases after pancreaticogastrostomy and 45% of cases with the dilated main pancreatic duct were symptomatic.^9)^ In a report by Murakami et al. that investigated the pancreatic duct diameter after pancreaticoduodenectomy, pancreatic duct dilation was observed in 55% of cases with pancreaticogastrostomy and 31% of cases with pancreaticojejunostomy. In terms of the anastomosis method, dilation was observed in 24% of cases with pancreatic duct mucosal anastomosis and 65% with the invagination method.^10)^ In cases of anastomotic stenosis, excluding pancreatic duct stenosis due to new tumor lesions or anastomotic recurrence, acute pancreatitis of the remnant pancreas is the most clinically problematic, which has been reported to occur in 6% of cases.^9)^

In the past, resection of the anastomotic site and reanastomosis with pancreatico-enterostomy were recommended for anastomotic stenosis,^1)^ but now endoscopic treatment is performed first, and surgical treatment is employed if conservative or endoscopic treatment is ineffective.^3)^ Endoscopic treatment involves endoscopically confirming the anastomosis using balloon dilatation or stent placement at the stenosis, and lithotomy is performed if pancreatic stones are present.

When the anastomosis is not identified, the main pancreatic duct of the remnant pancreas can be punctured under endoscopic ultrasound from the stomach, and a cannula can be introduced through the anastomosis to join the endoscope in the intestine (the “rendezvous” method).^2)^ Alternatively, if the “rendezvous” is difficult, EUS-PD alone can be performed.

In cases where endoscopic treatment is difficult, surgical treatment is indicated, and one of the surgical procedures is resection and reconstruction of the pancreatico-enterostomy.^1)^ This surgical procedure has the advantage that it can be performed using the same technique as pancreatico-enterostomy after ordinary pancreaticoduodenectomy. However, there are some disadvantages: the same elevated jejunum cannot be used, and a newly elevated jejunum may be necessary. In such cases, the length of the small intestine involved in digestion will be shortened, pancreatic function will be somewhat impaired due to additional pancreatic resection, and stenosis of the new pancreatico-enterostomy might recur if a similar reconstruction is performed under the same condition. Lateral (longitudinal) pancreaticojejunostomy is sometimes performed to prevent restenosis after surgery and has been reported to be effective.^11,12)^ Moreover, if there is room on the blind end side of the elevated jejunum, lateral pancreaticojejunostomy might be performed using the same limb of the jejunum.^12)^

The surgical procedure in our case was an extension of the endoscopic approach, in which the guidewire was led from the stomach to the anastomosis and introduced into the intestine (the “rendezvous” method) during laparotomy. While endoscopic incision of the obstructed anastomosis may be a feasible option in some cases, we chose the described surgical approach in this case due to the inability to clearly visualize the anastomosis endoscopically, the possibility of an occult fistula, and concerns about the risk of perforation. Unlike other surgical approaches involving re-anastomosis, this method has a low risk of pancreatic fistula and is thought to be useful in preserving pancreatic function. However, as the anastomosis is not performed directly, there is a disadvantage in that the treatment effect is inferior to that of reconstruction surgery. There have been few reports on surgical procedures for stenosis of pancreatico-enterostomy after pancreaticoduodenectomy, so further accumulation of cases will be necessary. This surgical “rendezvous” approach could serve as a surgical alternative method when endoscopic dilation is difficult.

## CONCLUSIONS

We report the case in which a surgical “rendezvous” was performed after EUS-PD for anastomotic stenosis of pancreatico-jejunostomy after pancreaticoduodenectomy. This procedure may be considered as one of the surgical approaches for managing stenosis.

## DECLARATIONS

### Funding

No funding was received.

### Authors’ contributions

MI drafted this manuscript; SM and MM critically revised the manuscript; MI, SM, SA, TM, MM, KK, AM, and MU were involved in treatment; and AM, TK, and MU reviewed the manuscript.

All authors read and approved the final manuscript.

### Availability of data and materials

The datasets used during the current study are available from the corresponding author on reasonable request.

### Ethics approval and consent to participate

Not applicable.

### Consent for publication

Informed consent was obtained from the patient for publication of this case report and any accompanying images.

### Competing interests

The authors declare that they have no competing interests.

## References

[1] Arai T Yabuki H Karasaki H Three cases of pancreaticogastrointestinal anastomosis reconstruction after pancreaticoduodenectomy (in Japanese). Nihon Rinsho Geka Gakkai Zasshi (Journal of Japan Surgical Association) 2000; 61: 1048–52.

[2] Takikawa T Kanno A Masamune A Pancreatic duct drainage using EUS-guided rendezvous technique for stenotic pancreaticojejunostomy. World J Gastroenterol 2013; 19: 5182–6. 23964156 10.3748/wjg.v19.i31.5182 PMC3746394

[3] Vanbrugghe C Campanile M Caamaño A Management of delayed stenosis of pancreatico-enteric anastomosis following pancreatoduodenectomy. J Visc Surg 2019; 156: 30–6. 30119964 10.1016/j.jviscsurg.2018.07.009

[4] Fujii T Sugimoto H Yamada S Modified Blumgart anastomosis for pancreaticojejunostomy: technical improvement in matched historical control study. J Gastrointest Surg 2014; 18: 1108–15. 24733259 10.1007/s11605-014-2523-3

[5] Boninsegna E Manfredi R Negrelli R Pancreatic duct stenosis: differential diagnosis between malignant and benign conditions at secretin-enhanced MRCP. Clin Imaging 2017; 41: 137–43. 27840266 10.1016/j.clinimag.2016.10.020

[6] Morgan KA Fontenot BB Harvey NR Revision of anastomotic stenosis after pancreatic head resection for chronic pancreatitis: is it futile? HPB (Oxford) 2010; 12: 211–6. 20590889 10.1111/j.1477-2574.2009.00154.x PMC2889274

[7] Joliat GR Allemann P Labgaa I Functional, biological, and radiological evaluation of the pancreaticojejunal anastomosis 1 year after pancreatoduodenectomy: a prospective study. Langenbecks Arch Surg 2023; 408: 326. 37606699 10.1007/s00423-023-03040-x PMC10444682

[8] Zarzavadjian Le Bian A Cesaretti M Tabchouri N Late pancreatic anastomosis stricture following pancreaticoduodenectomy: a systematic review. J Gastrointest Surg 2018; 22: 2021–8. 29980974 10.1007/s11605-018-3859-x

[9] Kurosaki I Hatakeyama K Kobayashi T Pancreaticogastrostomy: unreliable long-term pancreatic duct patency. Hepatogastroenterology 2003; 50: 545–9. 12749269

[10] Murakami M Kanji K Kato S Clinical influence of anastomotic stricture caused by pancreatogastrointestinalstomy following pancreatoduodenectomy. Surg Today 2017; 47: 581–6. 27631759 10.1007/s00595-016-1412-7

[11] Matsumoto I Kamei K Kawaguchi K Longitudinal pancreaticojejunostomy for pancreaticodigestive tract anastomotic stricture after pancreaticoduodenectomy. Ann Gastroenterol Surg 2021; 6: 412–9. 35634185 10.1002/ags3.12528 PMC9130871

[12] Demirjian AN Kent TS Callery MP The inconsistent nature of symptomatic pancreatico-jejunostomy anastomotic strictures. HPB (Oxford) 2010; 12: 482–7. 20815857 10.1111/j.1477-2574.2010.00214.x PMC3030757

